# Real-time intradermal continuous glucose monitoring using a minimally invasive microneedle-based system

**DOI:** 10.1007/s10544-018-0349-6

**Published:** 2018-12-06

**Authors:** Federico Ribet, Göran Stemme, Niclas Roxhed

**Affiliations:** Department of Micro and Nanosystems, KTH Royal Institute of Technology, SE-100 44 Stockholm, Sweden

**Keywords:** Diabetes, Continuous glucose monitoring system (CGMS), Microneedle, Electrochemical biosensor, Biomedical MEMS, Dermal interstitial fluid (ISF)

## Abstract

Continuous glucose monitoring (CGM) has the potential to greatly improve diabetes management. The aim of this work is to show a proof-of-concept CGM device which performs minimally invasive and minimally delayed *in-situ* glucose sensing in the dermal interstitial fluid, combining the advantages of microneedle-based and commercially available CGM systems. The device is based on the integration of an ultra-miniaturized electrochemical sensing probe in the lumen of a single hollow microneedle, separately realized using standard silicon microfabrication methods. By placing the sensing electrodes inside the lumen facing an opening towards the dermal space, real-time measurement purely can be performed relying on molecular diffusion over a short distance. Furthermore, the device relies only on passive capillary lumen filling without the need for complex fluid extraction mechanisms. Importantly, the transdermal portion of the device is 50 times smaller than that of commercial products. This allows access to the dermis and simultaneously reduces tissue trauma, along with being virtually painless during insertion. The three-electrode enzymatic sensor alone was previously proven to have satisfactory sensitivity (1.5 nA/mM), linearity (up to 14 mM), selectivity, and long-term stability (up to 4 days) *in-vitro*. In this work we combine this sensor technology with microneedles for reliable insertion in forearm skin. *In-vivo* human tests showed the possibility to correctly and dynamically track glycaemia over time, with approximately 10 min delay with respect to capillary blood control values, in line with the expected physiological lag time. The proposed device can thus reduce discomfort and potentially enable less invasive real-time CGM in diabetic patients.

## Introduction

Diabetes mellitus affects 400 million people worldwide and involves dangerous oscillations of the glucose levels in the body, caused by the lack of insulin production by the pancreas or by the faulty physiological response to this hormone. In order to avoid diabetes-induced complications such as retinal damage, cardiovascular diseases and neuropathies, careful glycaemia monitoring and management is essential (Bailes 2002). Traditionally, glucose monitoring is self-performed by patients using capillary blood extracted via finger pricking, typically two to five times per day. However, continuous glucose monitoring (CGM) has been shown to significantly improve the quality of diabetes treatment, providing continuous real-time measurements and a complete temporal overview (Dunn et al. 2018; Nathan et al. 1993). In addition, CGM can also reduce collateral effects of finger pricks, such as pain, infections, and sensory loss (Le Floch et al. 2008; Lodwig et al. 2014), as well as the patients’ discomfort and thus potentially improve their quality of life.

Continuous glucose monitoring systems (CGMS) have been developed in the last two decades. In particular, to perform minimally invasive glucose sensing, transdermal electrochemical sensors remain the most common and reliable solution (Bandodkar and Wang 2014; Heller and Feldman 2010; Vashist et al. 2011; Wang 2008). Moreover, several commercial CGM devices have been presented (Csöregi et al. 1995; Feldman et al. 2003; Heller and Feldman 2008; Rodbard 2016; Yoo and Lee 2010) and released to the market since the early 2000s. Invasiveness and cost are currently the main reasons for the low compliance and adoption of CGMS by diabetic patients. These commercial devices mostly consist of flexible sensing strips, approximately 6 mm long, inserted through the skin using retracting hypodermic needles. This approach makes their use relatively invasive, and their insertion painful. Additionally, due to their size, monitoring is performed in the hypodermis, which is not an ideal location for reliable and fast glycaemia tracking, due to its heterogeneity and the scarce local distribution of capillaries compared to the dermal region (Cengiz and Tamborlane 2009). In fact, interstitial fluid (ISF) is more abundant in the dermis, and dermal glucose concentration dynamics more closely follow oscillations in the blood (Groenendaal et al. 2008; Wang et al. 2005).

Therefore, microneedle-based CGMS are gaining attention because of their reduced invasiveness (Invernale et al. 2014; Miller et al. 2012, 2016b), resulting in a virtually painless insertion procedure and a reduced tissue trauma and inflammation (Ventrelli et al. 2015). The size of a transdermally implanted device affects the tissue trauma during insertion and the degree of inflammation over time, both of which cause an increased foreign body reaction that can be detrimental for long-term measurements (El-Laboudi et al. 2013; Wang et al. 2015). Moreover, microneedles shorter than 1 mm, which is the average depth of the dermis in the human forearm (Hwang et al. 2016), allow direct access to the dermal interstitial fluid. Finally, microneedle arrays for intradermal drug delivery, such as insulin injection, have previously been reported (Ita 2015; Larrañeta et al. 2016; Van Der Maaden et al. 2012; Roxhed et al. 2008), and this feature combined with microneedle-based CGM could enable a minimally invasive closed-loop system.

Current microneedle-based systems have shown issues in efficiently accessing the dermal interstitial fluid and performing real-time, cost-effective and reliable glucose monitoring. These issues include the need for complex interstitial fluid extraction mechanisms based on vacuum suction, microfluidic valves or liquid pre-filling (Gattiker et al. 2005; Miller et al. 2016a; Ricci et al. 2008; Vesper et al. 2006; Wang et al. 2005; Yu et al. 2012; Zimmermann et al. 2004), the risk of clogging of the needle opening during insertion or unreliable skin penetration (Chua et al. 2013), and the need for complex electrode functionalization procedures on non-flat surfaces (Sharma et al. 2016; Windmiller et al. 2011). Additionally, a significant measurement delay is typically introduced by the time required for the glucose molecules to diffuse from the interstitial fluid to the electrode area, typically situated in a compartment on the backside of the microneedle chip (Jina et al. 2014; Mukerjee et al. 2004; Strambini et al. 2015). The proximity of the sensing electrodes to the interstitial fluid in the skin is a key requirement to reduce the delay introduced by the skin-to-sensor diffusion time, a limiting factor in previous technologies. If this time is not minimized, the sum of the physiological lag time between blood glucose and interstitial fluid glucose concentrations, which is of the order of 4 to 12 min (Aussedat et al. 2000; Boyne et al. 2003), and the CGMS delay itself impedes real-time monitoring, and hence the possibility to take medical decisions, such as insulin delivery or hypoglycemia treatment, during the required time frame.

**Fig. 1:**
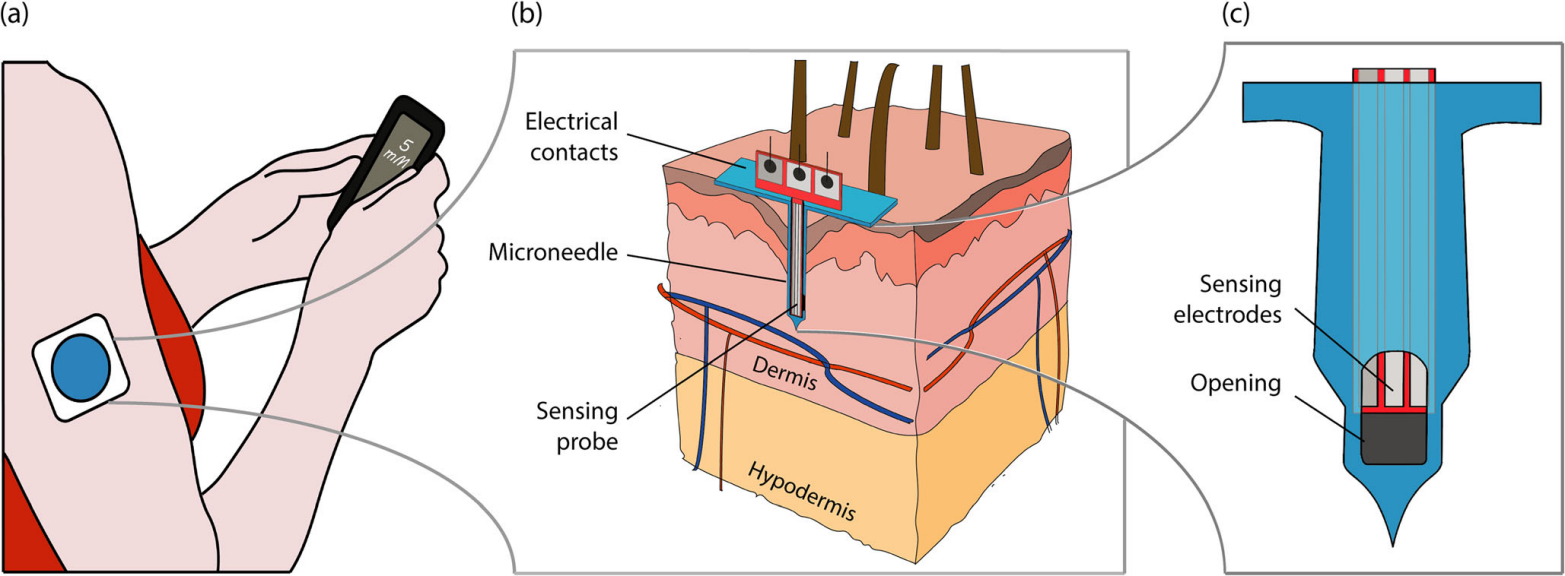
**a** Illustration of the typical implementation of a commercial CGM system. The focus of this work is specifically on the development, integration, and intradermal insertion of the sensing part of the CGM system. **b** Illustration of the cross-section of the developed CGM device inserted into the skin. The sensing probe is assembled inside a hollow microneedle lumen with the sensing electrodes facing the opening that allow diffusion from and to the dermal interstitial fluid. **c** Enlarged view of the three sensing electrodes facing the lateral opening of the microneedle

In this work we report a proof-of-concept prototype that combines the advantages of microneedle-based CGMS, in terms of reduced invasiveness, reduced tissue trauma and measurement location, with the *in-situ* plug-and-measure configuration of commercial CGMS. To achieve this, we realized and characterized an integrated system formed by a miniaturized sensing probe, incorporating a full three-electrode electrochemical cell, inserted in the lumen of a single hollow silicon microneedle. This system is designed to be fabricated using standard silicon microfabrication technologies and planar functionalization techniques. Moreover, the device does not require external actuators to extract the interstitial fluid. Instead, it relies purely on capillary action to fill the microneedle lumen volume, thus requiring only sub-nanoliter volumes of interstitial fluid to operate. The concept is illustrated in Fig. 1.

In our previous research, we have demonstrated the possibility to realize a sensor sufficiently small to fit inside a microneedle lumen, and hence in the dermal space, while preserving satisfactory stability, linearity, sensitivity, and selectivity *in-vitro* (Ribet et al. 2017, 2018). In this article, we characterize and demonstrate the possibility to perform *in-situ* minimally delayed and minimally invasive glucose monitoring in human skin *in-vivo*. Thus, the proposed integrated CGM device has the potential to improve comfort and treatment quality in patients affected by diabetes.

## Design, materials and methods

### CGMS design

The CGM device consists of two components: a miniaturized electrochemical sensing probe (70 × 700 × 50 μm^3^) and a 700 μm-long silicon microneedle. These two parts were independently microfabricated using standard MEMS fabrication techniques, and then assembled as a last step. The microneedle geometry was previously developed to have a sharp tip geometry for easy penetration and a side opening to avoid tissue clogging the lumen during insertion (Roxhed et al. 2007). The same microneedles were previously successfully used for insulin delivery (Roxhed et al. 2008), as well as intradermal injection of rabies vaccine in humans in a clinical study (Vescovo et al. 2017).

A key feature of the developed system with respect to previously proposed devices is related to the extreme sensor miniaturization. This enables measurement from the inside of the microneedle lumen and hence a minimal distance between the opening and the sensing electrodes, allowing, in turn, *in-situ* measurement purely by glucose diffusion. As previously discussed, for real-time monitoring, the time delay introduced by the diffusion of glucose molecules from the dermal interstitial fluid to the electrodes must be minimized. The time required for a glucose molecule to diffuse, e.g., 1 mm in a phosphate buffered saline solution, analytically calculated using Fick’s law, is approximately 17 min. The distance dermis-to-electrode should thus be kept in the range of tens to a few hundred micrometers to enable real-time monitoring, as in the design proposed here.

### Chemicals and instrumentation

Glucose oxidase (GOx, aspergillus niger VII), glutaraldehyde (GA, grade I, 25%), bovine serum albumin (BSA), d-(+)-glucose, and phosphate buffered saline (PBS, pH 7.4) were purchased from Sigma-Aldrich, Sweden. The *in-vitro* characterization was performed using a 0.01 M PBS measurement solution. To dispense the functional membrane solutions, a high-precision liquid dispenser (Ultimus V, Nordson EFD, UK) was used. Amperometric measurements were performed using a sub-picoampere resolution potentiostat (DY2011, Digi-Ivy, Inc., Texas, USA). A commercial 3 M Ag/AgCl reference electrode (REF321 Radiometer Analytical, Hach, Germany) was used as a reference during electrode preparation and to perform control tests. To perform glycaemia control tests in capillary blood, a commercial glucose meter was used (FreeStyle Lite, Abbott AB, Sweden).

### Sensor working principle

The developed sensor is a three-electrode electrochemical enzymatic glucose sensor, based on the transformation of glucose molecules into hydrogen peroxide by the enzyme glucose oxidase. The H_2_O_2_ concentration is then anodically detected at the surface of a platinum working electrode, biased with respect to an integrated iridium oxide (IrOx) pseudo-reference electrode (pseudo-RE) (Ribet et al. 2017; Waleed Shinwari et al. 2010), thus providing a current proportional to the initial glucose concentration, according to the reactions:

$$ \mathrm{Glucose}+{\mathrm{O}}_2\ \overset{GOx}{\to }\ \mathrm{Gluconic}\ \mathrm{acid}+{\mathrm{H}}_2{\mathrm{O}}_2 $$

$$ {\mathrm{H}}_2{\mathrm{O}}_2\kern0.50em \overset{+500\  mV\  vs. IrOx}{\to }{\mathrm{O}}_2+2{\mathrm{H}}^{+}+2{\mathrm{e}}^{-} $$

A counter electrode, also made of platinum, is responsible for closing the electrical circuit.

In our previous research we have demonstrated the possibility to realize an ultra-miniaturized sensor, with an overall sensing area footprint of less than 0.04 mm^2^, showing selectivity and sensitivity in line with ISO guidelines for glucose sensing, and long term stability. More details regarding the material choice and the characterization are reported elsewhere (Ribet et al. 2017). For fabrication simplicity, in this study we did not include the perm-selective membranes made of Nafion and polyurethane, partially sacrificing linearity range and interfering species rejection. Those layers, or equivalent alternative solutions, would be necessary for commercial products. However, we verified that these were not required to demonstrate the proof of concept discussed in this article *in-vivo*.

**Fig. 2:**
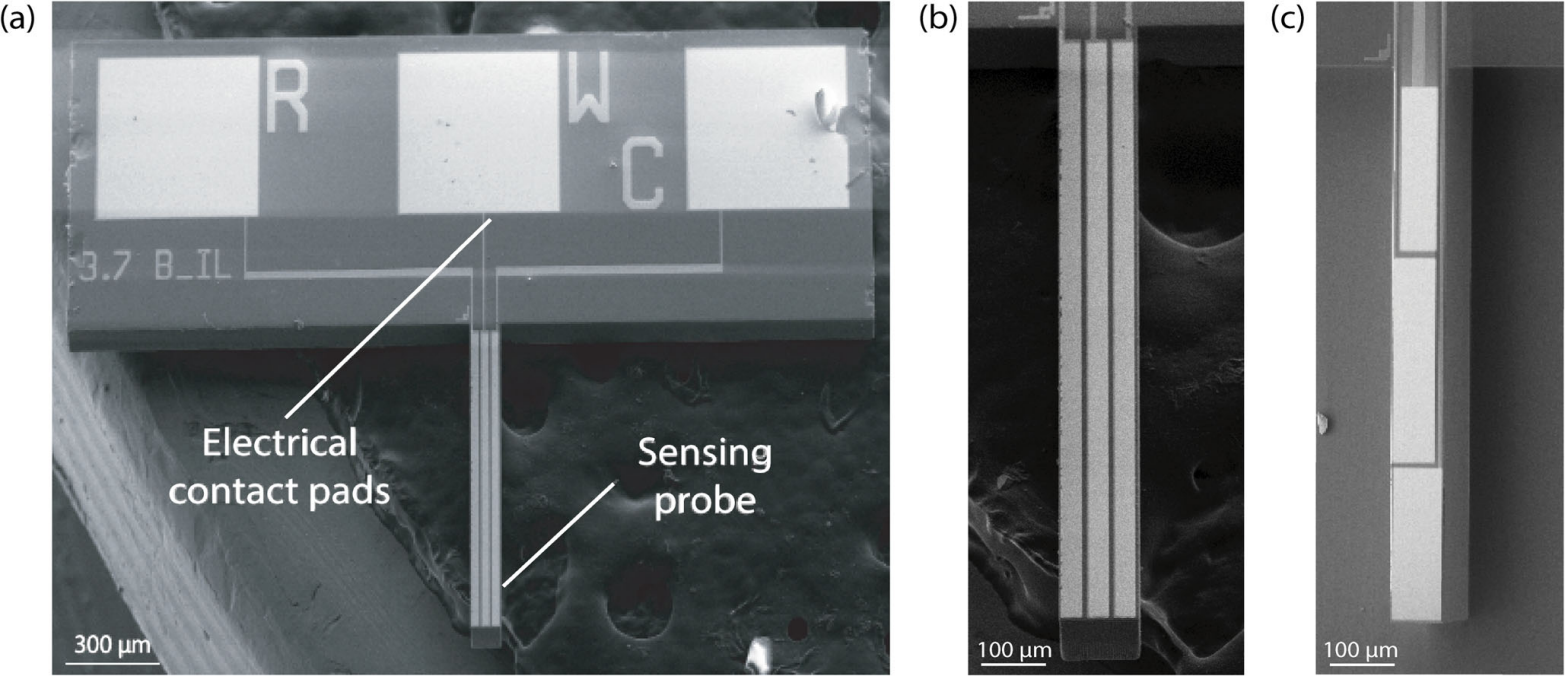
SEM top views of the sensors, before functionalization and assembly. **a** “T-shaped” sensor, with electrical contact pads on top and the three sensing electrodes on the probe at the bottom. **b** Closer view of the configuration with three long parallel electrodes, providing equal conditions at each electrode during measurement. **c** Alternative version with three vertically patterned electrodes, potentially enabling distinct functionalization of the different electrodes

Figure 2a shows a top-view scanning electron microscope (SEM) picture of the “T-shaped” sensor chip, formed by the miniaturized sensing probe and the electrical contact pad area. Two geometrical arrangements of the electrodes were realized: long parallel electrodes, as in Fig. 2b, and vertically stacked electrodes, as in Fig. 2c. The former geometry allows all three electrodes to be exposed to the same conditions in the lumen during measurement, which is especially important for *in-vivo* experiments; the latter allows for distinct functionalization of the three electrodes when dispensing the membrane solutions, potentially improving the sensor performance, as discussed in previous work (Ribet et al. 2017). Here, the latter configuration is only employed for microneedle *in-vivo* insertion and filling tests, while the former configuration is used for the amperometric experiments.

### Device fabrication and assembly

The sensing probes were realized using standard microfabrication techniques on a 300-μm-thick silicon substrate and were processed as follows. To insulate the substrate from the electrodes, a 1-μm-thick silicon dioxide layer was thermally grown. A platinum layer of 100 nm, patterned using lift-off lithography, was deposited to form electrodes and interconnections. 80 nm of iridium were then selectively deposited on the pseudo-RE locations, using lift off. Both metals were deposited on a 20-nm-thick titanium adhesion layer using electron beam evaporation. A 200 nm layer of PECVD silicon nitride was then deposited, and openings corresponding to electrodes and connection pads were formed by wet etching in buffered hydrofluoric acid, after photoresist patterning. To fit inside the microneedle lumen, the chips had to be singularized in the shape of thin probes. For this purpose, after photoresist patterning, 50-μm-deep trenches outlining the “T-shaped” sensor chip area were formed on the front side using deep reactive ion etching. Following this, the wafer was attached to a carrier substrate with an acetone-soluble adhesive and thinned from the wafer backside using dry silicon etching, after resist patterning with a geometry corresponding to the sensor area on the front side. Finally, the 50-μm-thin chips were released from the carrier wafer in acetone and thoroughly rinsed.

**Fig. 3:**
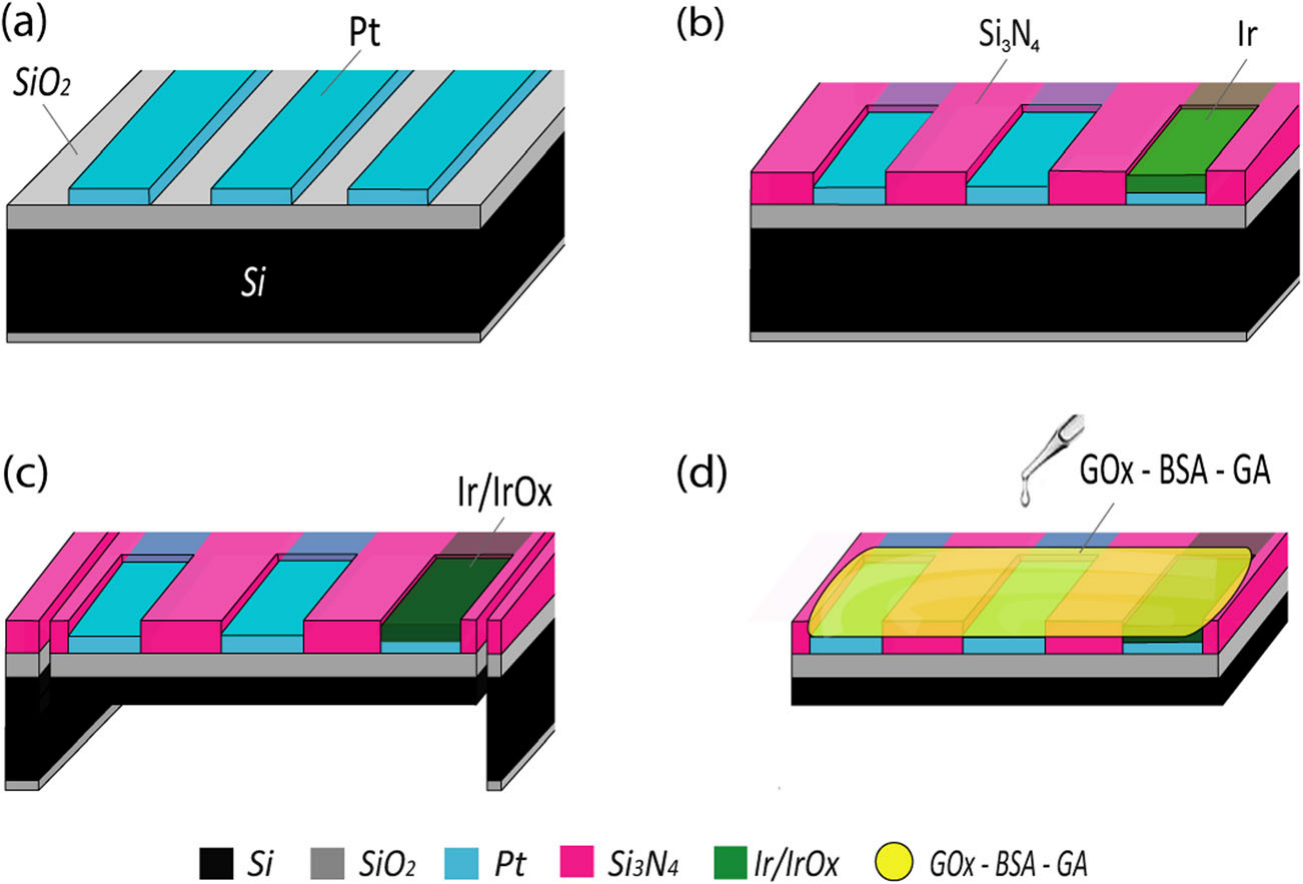
Illustration of the microfabrication steps to realize the sensor probe. **a** Platinum electrode deposition and patterning, **b** Iridium deposition and silicon nitride passivation of the interconnections, **c** front- and backside silicon etching to thin down and singularize the chips, and iridium pseudo-RE oxidation, **d** enzymatic functionalization of the electrodes with a precision liquid dispenser

Connection wires were attached to the sensor pads with conductive epoxy glue to allow interfacing with measurement instrumentation. To form an iridium oxide layer, cyclic voltammetry was used, by cycling 20 times the pseudo-RE potential in PBS solution between −0.75 V and + 0.9 V versus a Ag/AgCl reference electrode. To functionalize the electrodes, approximately 2 nL of an aqueous solution of 1% GOx and 1% BSA were dispensed using the precision liquid dispenser. To crosslink the enzymatic membrane, 2 nL of a 2% GA aqueous solution were then dispensed on top. Positional precision of the dispensed droplet was achieved by mounting the dispenser nozzle on a mechanical xyz-stage with micrometer positioners. The described microfabrication process steps are illustrated in Fig. 3.

**Fig. 4:**
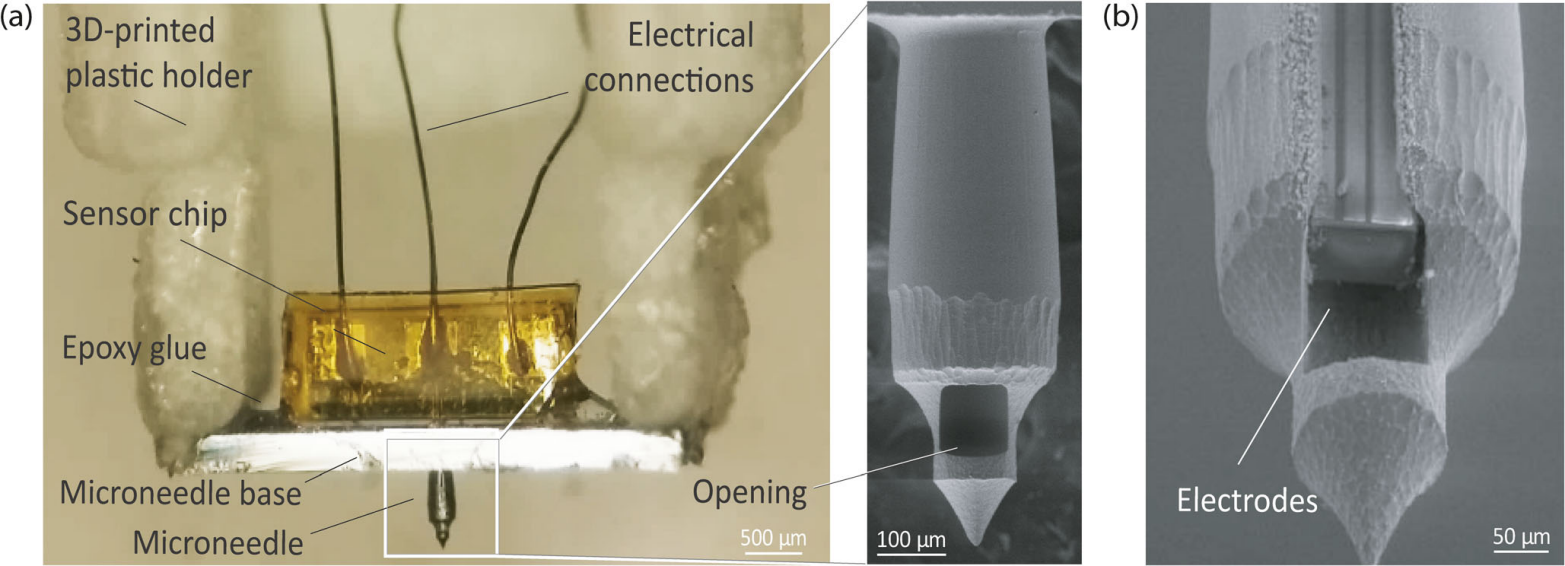
**a** Assembled CGM device, attached to the 3D printed plastic holder, with the sensor inserted in the microneedle lumen. Inset: SEM picture of the hollow silicon microneedle, with the side opening close to the sharp tip. **b** Close-up view of the microneedle opening. For visualization purposes, the opening has been enlarged by laser ablation to display the three-electrode sensor inside the microneedle lumen

The hollow silicon microneedles were fabricated with a sequence of isotropic and anisotropic dry etching steps on the front and back sides of a silicon wafer, as described elsewhere (Roxhed et al. 2007), providing the geometry shown in the SEM picture in the inset in Fig. 4a. Each microneedle is 700 μm long and the shaft at the widest point is 260 μm in diameter, whilst the lumen is approximately 90 μm in diameter.

The obtained sensor chip was inserted inside the microneedle lumen and fixated in position with epoxy glue at its edges, as shown in Fig. 4a. The device was then glued to a 3D-printed plastic holder, to allow it to be mounted on a custom-made insertion tool. The three-electrode probe inserted inside the microneedle lumen is shown in Fig. 4b.

### *In-vivo* insertion and characterization procedure

Despite the needle geometry, prolonged microneedle insertion was not consistently achieved for the time needed for the experiments by simply placing the device onto the skin and manually pressing. In fact, the insertion speed plays a fundamental role in piercing the skin and achieving deep and reliable insertion (Verbaan et al. 2008). This is due to the ability of the skin to elastically deform under the applied pressure (Crichton et al. 2011). In particular, in CGM applications, proper insertion is extremely critical due to the need for prolonged measurements, ranging from hours to days, as opposed to microneedle-based delivery, which is instead performed within minutes. Thus, in order to achieve proper skin penetration, the CGM device insertion was performed using a custom-made insertion tool, consisting of an armrest attached to a spring-loaded mechanism (see Fig. 7a). The insertion tool generated an initial force of 5 N at maximum spring contraction, and a residual force of 0.5 N when the device was in contact with the skin. The calculated generated penetration velocity was approximately 3 m/s.

The CGM devices were then characterized *in-vivo*, in healthy Caucasian human volunteers in accordance with ethical standards (IRB approval: EPN Stockholm, Dnr. 2015/867–31/1) and upon informed consent, to assess proper functioning. The forearm was chosen as the measurement location to perform characterization. There, the skin is easily accessible, the epidermis relatively thin and easy to penetrate, with an average thickness of 74 μm (Hwang et al. 2016), and the 1-mm-thick dermal region can be reliably reached with 700 μm long microneedles (Coulman et al. 2011; Kalluri et al. 2011). After insertion, the CGM device was allowed to stabilize for 20 min under biasing conditions (+0.55 V), before starting the measurement. The *in-vivo* characterization was based on the so-called glucose tolerance test in which the subject, starting under fasting conditions, intakes a sugar dose while the glycaemia is measured over time until it returns to a normally-low level. For this test 35 g of sugar, of which 10 g of pure dextrose, were ingested by the subject while monitoring the glucose concentration curve over time with the presented CGM device. Finally, in order to verify correct device functioning and lag time, the readout was compared to capillary blood glucose values regularly obtained with the commercial device via finger pricks every 10 min.

## Experimental results and discussion

**Fig. 5:**
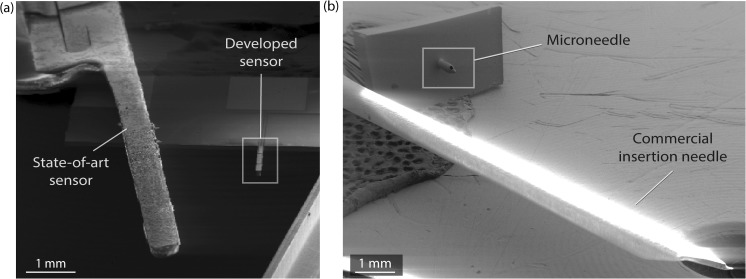
SEM image comparing our CGMS to a commercial state-of-art device (Abbott Freestyle Libre). **a** Comparison of the sensing probe with the 50-fold larger commercial sensor. **b** Comparison of our silicon microneedle with the commercial CGMS insertion needle

A volumetric implant reduction of nearly 50 times with respect to state-of-art devices was achieved, making the presented CGM device the smallest *in-situ* intradermal CGMS reported to date. Figure 5a shows an SEM picture providing a visual comparison between the presented sensor and a state-of-art commercial sensor (Freestyle Libre, Abbott AB, Sweden). Figure 5b shows the presented microneedle and the insertion needle of the same commercial CGMS providing a comparison in size, and thus an idea of the invasiveness and generated tissue trauma upon insertion in the skin.

### *In-vitro* CGM device characterization

The *in-vitro* stability and sensitivity of the miniaturized sensor alone were previously thoroughly characterized (Ribet et al. 2017), showing more than 4 days of iridium oxide open circuit potential stability in PBS, and a sensitivity of 1.5 nA/mM in the linear range. The stability of the enzyme was verified both during continuous operation, for several hours, and after storage up to 3 months.

To characterize the sensors integrated with the microneedle chips, the microneedle tip was positioned in contact with PBS solution, causing spontaneous filling of the lumen with liquid by capillary action. A + 0.55 V bias was applied to the working electrode with respect to the integrated pseudo-RE, and the sensor was then allowed to stabilize for few minutes. Once the current reached a stable background level, aliquots of glucose were sequentially added to the magnetically stirred PBS solution to obtain 50 mg/dL concentration steps in the measurement solution.

**Fig. 6:**
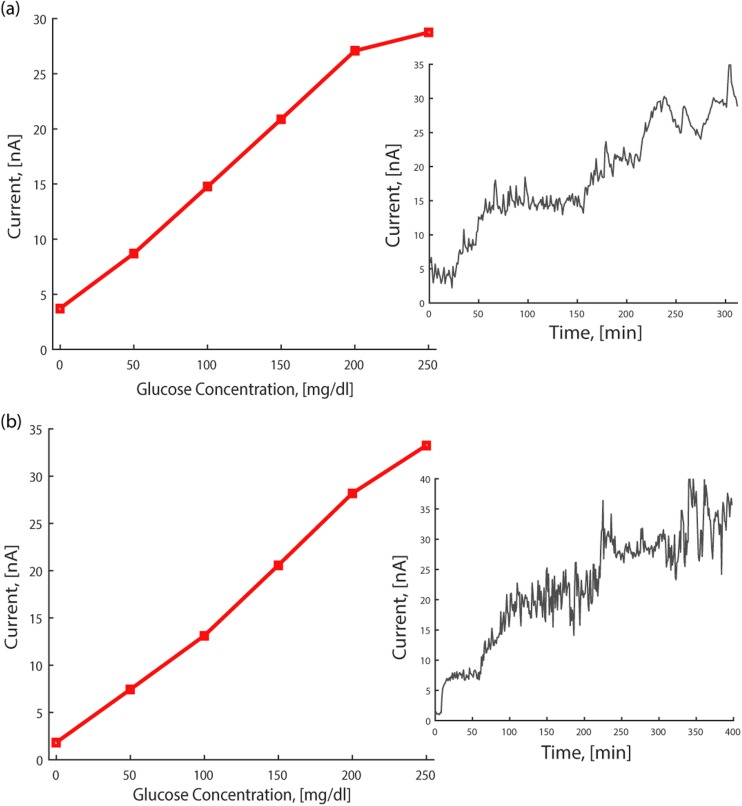
Characterization of the CGM device *in-vitro* in PBS solution. **a** Plot of current versus glucose concentration, using the integrated pseudo-RE, with a bias of +0.5 V. Inset: chrono-amperometry, showing the current at incremental concentration steps of 50 mg/dL each, over a period of 5 h to assess both functionality and stability over time. **b** Same experiment, but using an external commercial Ag/AgCl reference electrode and a bias of +0.6 V, to compare functionality and long-term stability of the integrated IrOx pseudo-reference electrode with a standard reference electrode. Inset: related chrono-amperometry

Figure 6 reports the amperometric characterization of the sensor. Figure 6a shows, for the integrated CGM device, the measured current with respect to the glucose concentration, while the inset in Fig. 6a shows the chrono-amperometric measurement. To assess the stability of the embedded iridium oxide pseudo-RE, the amperometric response of the same sensor was recorded while using an external macroscopic commercial Ag/AgCl reference electrode. The result is reported in Fig. 6b, and shows negligible performance differences with respect to Fig. 6a, thus confirming satisfactory pseudo-RE stability during operation inside the microneedle lumen.

Due to the absence of the perm-selective polyurethane layer, the obtained sensitivity is 2.44 nA/mM, almost double the value previously reported (Ribet et al. 2017). For the same reason, the measurement range, limited by the current saturation level, is halved and reached at a glucose concentration of approximately 200 mg/dL. This still provides a sufficient range to perform the desired *in-vivo* glucose tolerance test in healthy volunteers, as well as to measure minimum concentrations that would trigger insulin delivery need in diabetic patients. As previously demonstrated, by using a porous membrane such as polyurethane, the measurement range could be extended up to 500 mg/dL to match commercial blood glucose meters.

The effect of placing the probe in the lumen on the response time was also assessed. As previously discussed, to perform continuous real time monitoring, it is fundamental that the delay introduced by the sensor and the entire system is negligible with respect to the physiological concentration changes in the body. The response time was quantified as the time required to reach 90% of the average current value at a given concentration, after an increment in concentration of 50 mg/dL. On average the response time was 315 s for the integrated device and 300 s for the sensor alone, demonstrating that the delay introduced by the microneedle embodiment is negligible with respect to the sensor response time itself. Moreover, this translates into a maximum sensing variation rate of 9.5 mg/dL/min, which is well above the typical maximum physiological glucose variation rate (3 mg/dL/min) (Dunn et al. 2004), even allowing tracking of extreme glycemic variations. Hence, we have shown that the presented CGM device potentially enables minimally delayed real time continuous glucose monitoring under relevant physiological conditions.

### *In-vivo* CGM device characterization

The previously developed microneedles showed satisfactory robustness during penetration and sensing. No microneedle was observed to be damaged during *in-vivo* characterization, even when increasing the insertion speed up to 6 m/s.

**Fig. 7:**
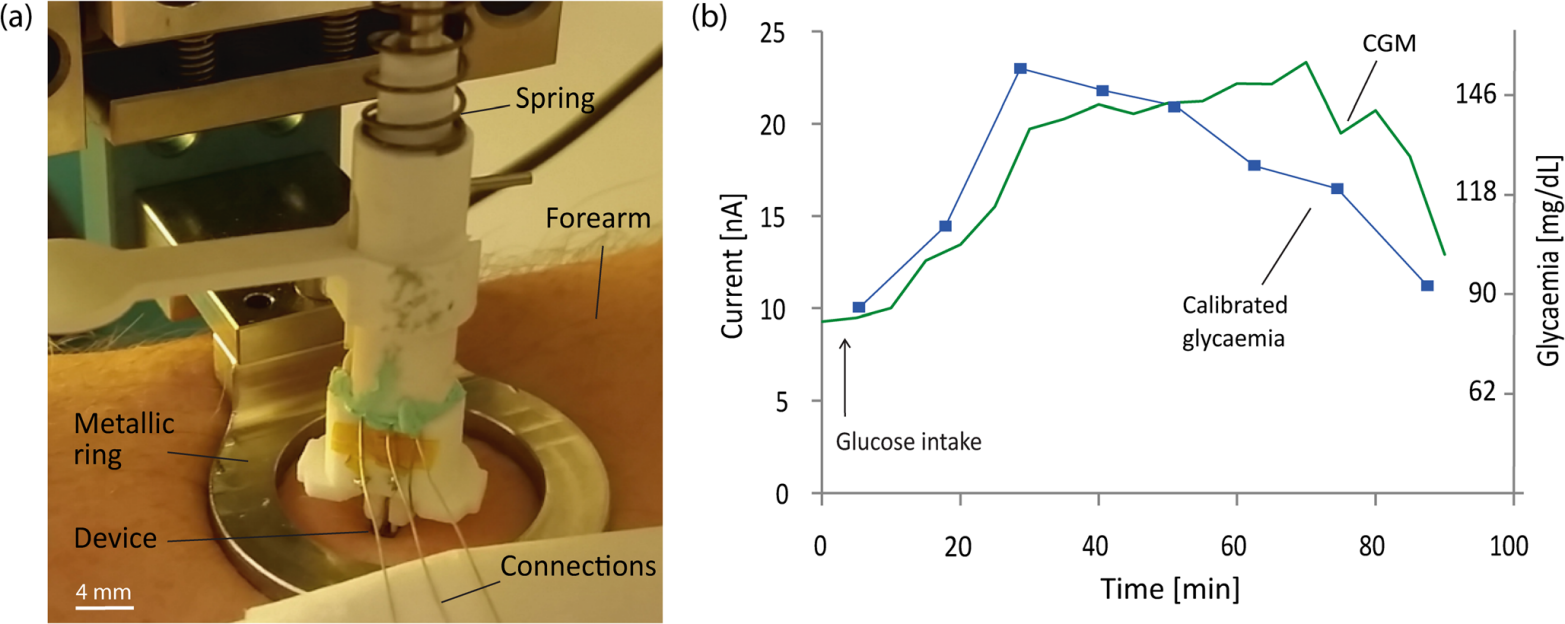
**a** Insertion setup. The microneedle chip is attached to a 3D-printed holder connected to a spring-loaded mechanism. A metal ring keeps the skin and the arm in position during the measurement. The wires are then connected to the potentiostat to perform the amperometric characterization. A closer view of the device is shown in Fig. 4a. **b** Glucose tolerance test results, performed in the human forearm. The CGM data obtained with the presented device are compared with blood glucose values to evaluate their correctness and lag time in tracking glycaemia, after glucose intake by the volunteer

To verify correct insertion and passive filling of the microneedle lumen with interstitial fluid, the resistance between two different sensor electrodes, as well as between each of the electrodes and an external electrode applied on the forearm skin, was measured. Successful insertion was verified by a distinct reduction in resistance, similar to the one obtained in pure PBS solution, between 1.5 MΩ and 2.5 MΩ. Penetration proved to be consistent using the spring-loaded applicator (Fig. 7a) and the given parameters. To prove complete filling of the lumen after insertion, the same test was also performed with the second electrode geometry, consisting of three vertically aligned electrodes (Fig. 2c). Complete filling was demonstrated by measuring an equal resistance reduction at the top electrode, positioned close to the top of the lumen, and at the bottom one, positioned close to the opening towards the skin. The calculated volume necessary to fill the microneedle lumen is less than 1 nL.

In this study, the insertion tool helped to keep the CGM device in position during characterization, but completely prevented the motion of the subject as well as conformal motion of the device with the skin. Notably, loss of insertion was the main failure mode of the device over time. Hence, to reduce the risk of microneedle ejection from the skin over time due to skin relaxation and elastic deformation, a thin adhesive layer was applied under the device base to fixate it to the skin. Although outside of the scope of this study, it would be conceivable to have an independent adhesive patch to keep the device in place after insertion, as is typically done in most commercial products and as illustrated in Fig. 1a. Further to improving prolonged insertion robustness, this would also allow longer term characterization *in-vivo*. Currently, measurements are limited to approximately 2 hours because of these mechanical limitations.

The amperometric *in-vivo* performance was tested on a human forearm under the same electrochemical conditions used for the *in-vitro* measurements in section 3.1, by performing the previously described glucose tolerance test. Figure 7b shows the current reading from our CGM device compared with the blood glucose data collected via finger pricks. A two-point calibration is used to compare the current readout of our sensor with the values in mg/dL provided by the commercial device. The CGM curve clearly follows the shape of the glycaemia curve, with only approximately 10 min of lag time, in line with the expected physiological lag time between glycaemia and glucose levels in the dermal interstitial fluid (4–12 min). Such delay can be considered acceptable and potentially further mitigated by smart modeling and data trend analysis (Facchinetti et al. 2013). This result confirms, as a proof-of-concept, the possibility to perform minimally invasive, correct and minimally delayed glycaemia tracking in the dermal interstitial fluid with our CGM device, without the need for extraction mechanisms or liquid pre-filled systems. However, these studies are performed in a laboratory under controlled conditions, i.e. without end-user-induced usage errors and without performing measurements under mechanical stress, such as during sport exercise or under vibrations. Before this technology can be marketed and obtain regulatory validation, robustness and correct operation of the system under mechanical stress, as well as correct prolonged operation *in-vivo* over several days, would need to be assessed. As previously mentioned, a patch-like system needs to be developed to reliably perform these assessments. Nevertheless, the obtained miniaturization may represent a major step forward in terms of user compliance due to the painless insertion, and an improvement in the quality of the obtained CGM data due to the ideal measurement location. Finally, the 50-fold reduction in terms of sensor dimensions and the system simplicity have the potential to reduce the production cost, which currently represents a hurdle for users and healthcare systems.

## Conclusions

We presented the first integrated system designed to perform *in-situ* minimally invasive continuous glucose monitoring in the dermal region with minimal delay with respect to glycaemia variations. The 50-fold miniaturization compared to state-of-art products makes this system significantly less invasive than commercial CGMS. Moreover, it enables access to the interstitial fluid in the dermis, which is a better monitoring location with respect to the hypodermis, where commercial devices are forced to measure because of their larger size. Passive interstitial fluid extraction was achieved by capillary filling of the microneedle lumen, without the need for an external actuator or of liquid pre-filling procedures, providing a simpler and more cost-effective solution compared to state-of-the-art microneedle-based systems. Amperometric tests *in-vitro* and on human forearm *in-vivo* provided proof of correct operation of the presented prototype, with satisfactory sensitivity and a measurement delay in line with the physiologically expected value. This system can therefore potentially enable minimally invasive, simple, fast and reliable CGM in the dermal interstitial fluid of patients affected by diabetes.
